# Whole Genome and Transcriptome Sequencing of a B3 Thymoma

**DOI:** 10.1371/journal.pone.0060572

**Published:** 2013-04-05

**Authors:** Iacopo Petrini, Arun Rajan, Trung Pham, Donna Voeller, Sean Davis, James Gao, Yisong Wang, Giuseppe Giaccone

**Affiliations:** 1 Medical Oncology Branch, National Cancer Institute, Bethesda, Maryland, United States of America; 2 Genetics Branch, National Cancer Institute, Bethesda, Maryland, United States of America; Institute of Molecular Medicine, Taiwan

## Abstract

Molecular pathology of thymomas is poorly understood. Genomic aberrations are frequently identified in tumors but no extensive sequencing has been reported in thymomas. Here we present the first comprehensive view of a B3 thymoma at whole genome and transcriptome levels. A 55-year-old Caucasian female underwent complete resection of a stage IVA B3 thymoma. RNA and DNA were extracted from a snap frozen tumor sample with a fraction of cancer cells over 80%. We performed array comparative genomic hybridization using Agilent platform, transcriptome sequencing using HiSeq 2000 (Illumina) and whole genome sequencing using Complete Genomics Inc platform. Whole genome sequencing determined, in tumor and normal, the sequence of both alleles in more than 95% of the reference genome (NCBI Build 37). Copy number (CN) aberrations were comparable with those previously described for B3 thymomas, with CN gain of chromosome 1q, 5, 7 and X and CN loss of 3p, 6, 11q42.2-qter and q13. One translocation t(11;X) was identified by whole genome sequencing and confirmed by PCR and Sanger sequencing. Ten single nucleotide variations (SNVs) and 2 insertion/deletions (INDELs) were identified; these mutations resulted in non-synonymous amino acid changes or affected splicing sites. The lack of common cancer-associated mutations in this patient suggests that thymomas may evolve through mechanisms distinctive from other tumor types, and supports the rationale for additional high-throughput sequencing screens to better understand the somatic genetic architecture of thymoma.

## Introduction

Thymomas are rare mediastinal tumors with heterogeneous histological features and clinical behavior. According to the most recent WHO classification, thymomas are grouped into five subcategories (A, AB, B1, B2, B3), depending on cancer cell shape, degree of atypia and number of intratumoral thymocytes [1]. Surgery represents the mainstay of treatment of thymic epithelial tumors, and survival is strongly influenced by the stage of the disease and the completeness of tumor resection [1]. Metastatic and non-resectable thymic epithelial tumors are candidates for systemic therapy [2]. Although, combination chemotherapy is able to induce substantial tumor shrinkage of variable duration, it is usually not curative in patients with metastatic disease [2]. Molecular targeted drugs have become available for treatment of several cancer types, but the rational development of target-specific drugs for thymic epithelial tumor therapy has been delayed because the molecular aberrations of thymic epithelial tumors are largely unknown [2].

Among thymomas, type B3 displays the most aggressive behavior, with 10-year survival rates ranging from 50–70% [1] Microscopically, medium size round or polygonal epithelial tumor cells with slight atypia compose B3 thymomas; these cells are mixed with a minor component of intraepithelial thymocytes. Usually, neoplastic cells are polygonal with round or elongated nuclei often folded or grooved [1]. At the diagnosis, B3 thymomas are commonly not encapsulated and present an infiltrative border with extension to the mediastinal fat and the surrounding organs [1]. Therefore, invasive B3 thymomas frequently require preoperative treatment in order to reduce tumor extension and increase resectability. After surgery, 15–17% of B3 thymomas develop a local recurrence and 20% distant metastases [1], [3]. Comparative genomic hybridization (CGH) studies demonstrated a more abnormal karyotype in B3 than in other thymomas [4], [5], whereas to the best of our knowledge, no information is available regarding gene mutations in this subtype of tumors [2].

Next generation sequencing technologies offer a new prospective in the study of the cancer genome, allowing a genome-wide characterization of tumor specific mutations. Among others, the whole genome sequence of lung, breast, colon, and liver cancers have been reported [6], [7], [8], [9]. These frankly aggressive tumors present multiple mutations and a complex karyotype with a variable number of translocations. It is of interest to extend these studies and include low to moderate malignant types of tumors such as thymomas. Here we describe the characterization of thymoma specific mutations from the complete genome and transcriptome sequencing of a case of B3 thymoma.

## Materials and Methods

National Cancer Institute Institutional Review Board (Bethesda, MD) approved this study (ClinicalTrials.gov ID: NCT01306045) that was conducted in agreement with the Declaration of Helsinki. The patient gave its written informed consent.

### DNA and RNA Extraction from Frozen Material

Immediately after the resection, tumor fragments were snap frozen in liquid nitrogen and stored at −180°C. A frozen tumor sample was embedded in optimal cutting temperature compound (OCT) and 8 µm slides were cut. The slides were stained with Haematoxylin and Eosin and a pathologist confirmed the presence of >80% of tumor cells. The selected tumor region was macrodissected. Tumor DNA and RNA were extracted from the same frozen specimen using All prep RNA/DNA Mini kit (Qiagen, Valencia CA), according to vendor instructions. Patient’s blood was collected in 5 ml EDTA tubes and stored at −80°C. Normal DNA was extracted from whole peripheral blood using QIAamp DNA blood maxi kit (Qiagen), according to the company’s protocol.

### Whole Genome Sequencing

15 µg of genomic DNA from tumor and normal samples were used for whole genome sequencing. Complete Genomics Inc. (CGI, Mountain View, CA) accomplished the massively parallel short-read sequencing using a probe-anchor ligation chemistry combined with a patterned nanoarray-based platform of self-assembling DNA nano-balls [10]. CGI performed the alignment of reads to the human reference genome (NCBI Build 37) using a local de novo assembly. Variant calls were generated as previously described [10]. Briefly, the paired-end reads, with a peculiar gapped structure, were aligned to the reference genome in a two-step process. First, both extremities of the read were independently mapped to the reference genome: for each extremity, multiple possible locations were indexed. Subsequently, the proper read position was determined using local alignment of the opposite extremity at approximately the mate-pair distance. Variant calls were investigated at those locations that most likely differ from the reference, through the best-fit assembly of the reads mapped to the suspected region. Finally, a custom software suite, employing both Bayesian and de Bruijn graph techniques, yielded diploid genotype at each genomic location with associated variant quality scores. Somatic (tumor specific) variants were called across the whole genome using CallDiff software, included in CGA tools v1.3.0 package that is available from CGI website (http://cgatools.sourceforge. net/docs/1.3.0). CallDiff returned candidate somatic variations associated with a confidence score: the somatic score. A threshold score of 0.1 was used to filter the high confidence mutations reported in this article. This threshold was selected on the basis of previous reports [9], [11] and on the best fit of our experimental results obtained sequencing 104 loci by Sanger method. Single Nucleotide Variations (SNVs) with a somatic score >0.1 presented a specificity of 60% and a sensitivity of 94%. Somatic variants were annotated using Varscan for their effect on transcripts, their presence in dbSNP131 (http://hgdownload.cse.ucsc.edu/goldenPath/hg18/database/snp131.txt), in 1000 Genomes Project and in variants reported in the public database available from Complete Genomics website. Moreover, Sorting Tolerant From Intolerant (SIFT) (available at http://sift.jcvi.org) and Polyphen2 (available at http://genetics.bwh.harvard.edu/pph2) scores were determined to assess potential impact of the predicted SNVs on the structure of the translated proteins.

### Transcriptome Sequencing

RNA quality was tested using RNA 6000 nano kit (Agilent) and run on Agilent 2100 Bioanalyzer. The tumor sample RNA had a RNA integrity number >8 and passed our quality threshold for transcriptome sequencing. Libraries were constructed from 3 µg of total RNA using TruSeq RNA Sample Preparation Kits v2 (Illumina) according to vendor’s instructions. Briefly, enrichment for mRNA was carried out using polyA-tail capture. The RNA was chemically fragmented and converted into single strand cDNA using random hexamer priming. Then, for each fragment the complementary sequence was generated. An adenosine base was added to each end of the fragments and adaptors were ligated with a T base overhang on 3′ end. These products were denatured and amplified on a Cluster Station (Illumina). Paired-end reads of 101 bases were generated using HiSeq 2000 (Illumina) on a single line of the sequencer’s flow cell. The row sequence reads in FASTQ format were assessed for quality using FASTQC. Sequences were mapped to human genome (NCBI Build 37) using GSNAP (http://research-pub.gene.com/gmap/) and deduplicated by Picard (http://picard.sourceforge.net/). Gene expression was estimated using Cufflinks (University of California Berkeley; http://cufflinks.cbcb.umd.edu/).

### Confirmation of Candidate Mutations

Tumor specific non-synonymous, splicing site and miRNA loci mutations, not previously described as single nucleotide polymorphism (SNP), were selected for confirmation using conventional Sanger sequencing, independently from their somatic score. SNPs were excluded if listed in 1000 Genomes Project [12], in dbSNP131 [13] or within the public available dataset from Complete Genomics website, independently from their prevalence in different races. Four somatic mutations confirmed using Sanger sequencing but with a somatic score <0.1 were also included in tier 1. For confirmation of SNVs and insertion/deletions (INDELs), primers were designed across the mutation sites using CLC genomics workbench 4 and tested for hairpin and heteroduplex formation using Oliogo 6.8 Carbon (MBI, Cascade, CO). All designed primers were tagged with M13 sequence. PCR Supermix High Fidelity (Invitrogen) was used for PCR amplification that was automated on Veriti® 96-Well Thermal Cycler (Applied Biosystems) according to the following steps: step 1: 94°C for 10 minute; step 2: 35 cycles of 94°C for 30 s, 60°C for 45 s and 72°C for 45 s; step 3: 72°C for 7 minutes. PCR products underwent ExoSAP-IT® (USB, Cleveland, OH) purification. The purified products were directly sequenced using BigDye terminator v3.1 cycle sequencing kit (Applied Biosystems) and 3730×l DNA Analyzer (Applied Biosystems). Candidate mutations were evaluated in the sequence of tumor and normal DNA using Macvector (MacVector, Cary, NC). Similarly, candidate junction sequences were confirmed.

### Array Comparative Genomic Hybridization

The labeling of tumor and reference DNA was performed using Genomic DNA ULS labeling kit (Agilent), according to the vendor’s instructions. The DNA was hybridized on SurePrint G3 Human CGH Array 180K (Agilent). The array was scanned on a laser-based microarray scanner (Agilent), and the data were extracted and normalized using Feature Extraction 10.5 (Agilent). CN profile was inferred using Rank Segmentation algorithm and Nexus 6 (Biodiscovery Inc., El Segundo, CA, USA).

## Results

### Clinical History

**Figure 1:**
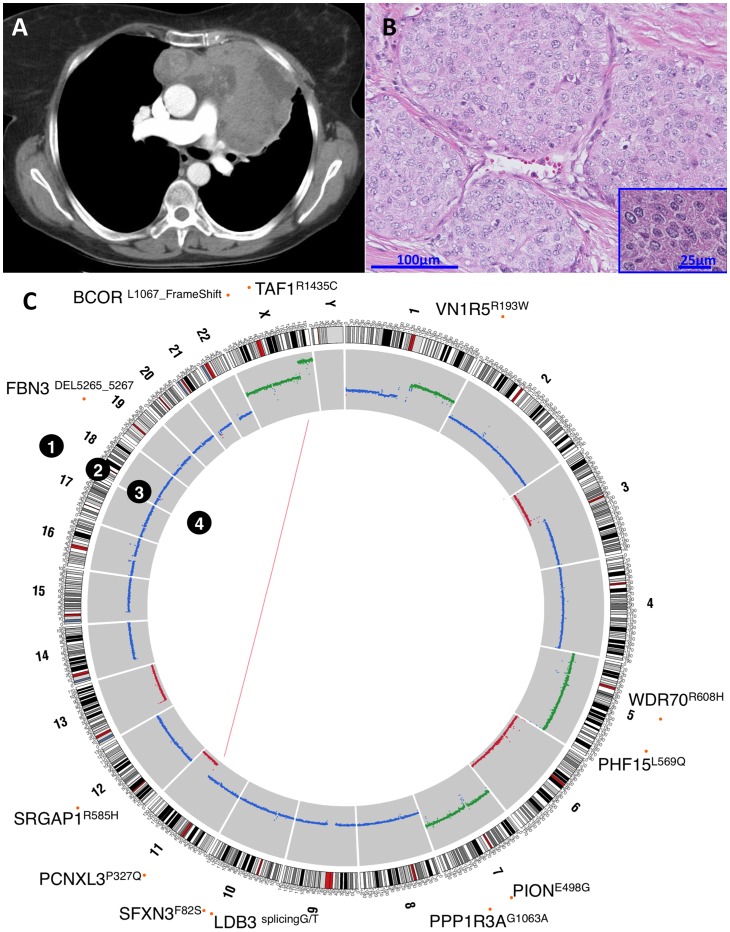
Features of the B3 thymoma. (A) Preoperative Chest CT-scan showing a mediastinal mass protruding in the left hemithorax. (B) Haematoxylin and Eosin staining of postoperative tumor section showing the lobular aspects typical of B3 thymomas, the almost complete absence of intratumoral thymocytes and the presence of cancer cells characterized by polygonal shape and round nuclei. (C) Whole-genome sequencing results showing (1) Sanger sequencing-confirmed SNVs and INDELs, (2) reference genomic coordinates, (3) copy number gain (green) and loss (red), and (4) structural variations.

In April 2007, a 55 year-old Caucasian female presented with shortness of breath and fatigue. A computed tomography (CT) revealed a left sided anterior mediastinal mass measuring 12.3×7.9 cm infiltrating the pericardium, the diaphragm and the left lung but without evidence of distant metastases. The patient was taken to the operating room for surgical resection with curative intent but during the procedure the tumor was judged unresectable and only a biopsy was performed. The pathological diagnosis was stage III WHO subtype B3 thymoma. She did not have any paraneoplastic syndrome associated with the disease. The patient received 6 cycles of cisplatin, doxorubicin, vincristine and cyclophosphamide (ADOC regimen) and achieved a 30% reduction in the size of the mediastinal mass. Five months later, the patient had disease progression and received an ifosfamide-based regimen with no response after three months. Thereafter, she received prednisone therapy, initially at 60 mg per day followed by a tapering dose over a period of 6 months and achieved disease stabilization. In March 2009, she was referred to the National Cancer Institute (NCI; Bethesda, MD), where a CT scan (Figure 1A) showed no evidence of disease progression with the mediastinal mass measuring 11×6 cm. The patient was evaluated by thoracic surgery and deemed to have resectable disease. She then underwent mediastinal exenteration of the thymoma, en bloc left intrapericardial pneumonectomy, resection of part of the left chest wall, mediastinal lymph node dissection, resection of a solitary pleural metastasis at the dome of the left diaphragm and reconstruction of the pericardium and chest wall without evidence of macroscopic residual disease. The pathology report confirmed the diagnosis of B3 thymoma infiltrating the pericardium, lung and the chest wall but with negative bronchial and surgical margins (Figure 1B). The patient refused adjuvant radiation therapy and had no evidence of disease for 26 months. Subsequently she developed fatigue, dyspnea (New York Heart Association grade 3) and chest pain associated with exertion and relieved by rest. Cardiac evaluation revealed no evidence of coronary artery disease, a left ventricle ejection fraction of 55% and the presence of soft tissue masses suggestive of disease recurrence. A CT scan of the heart showed tumor encasing the left main coronary artery and adjacent to the right coronary artery. Soft tissue was also seen along the right side of the heart and suspicious masses were seen along the left hemidiaphragm. An MRI of the heart showed tumor masses invading the left atrium and protruding into the left atrial cavity with additional invasion through the interatrial septum. The patient was evaluated by thoracic surgery and deemed to have unresectable disease at this stage. She was then treated with radiation therapy to the cardiac root mass, utilizing IMRT modality with 6MV photons to a total dose of 6300 cGy in 180 cGy fractions resulting in resolution of symptoms and a decrease in the size of intra- and para-cardiac masses. Currently, nine months after completion of radiation therapy, the patient continues to do well without any further evidence of disease progression.

### Whole Genome and Transcriptome Sequencing

Whole genome sequencing was accomplished mapping 222.2 and 234.53 Gb of sequences against the reference genome (NCBI Build 37) for tumor and normal DNA, respectively. 3×10^9^ and 3.17×10^9^ mapped reads allowed an average coverage of 78× and 82× in tumor and normal DNA, respectively. In tumor and normal, the sequence of both alleles was determined in more than 95% of the reference genome. A total of 3,096,049 SNVs were observed in tumor and 3,314,611 in normal DNA. Because the number of SNPs was higher in normal than in tumor DNA, we compared the number of SNPs in chromosomes affected or not by Copy Number (CN) loss. Chromosomes without CN loss presented a similar number of SNPs in normal and tumor DNA (average 2% more SNPs in normal DNA with a standard deviation of 2%). On the contrary, chromosomes affected by CN loss presented 24% more SNPs in normal than in tumor DNA (standard deviation 11%). Consequently, chromosomes with CN loss of tumor DNA had significantly less SNPs (p = 0.0001). Similarly, a larger number of INDELs were observed in normal DNA (Table 1). Somatic mutations were determined by comparing variation calls in tumor and normal DNA using CallDiff. The total number of candidate somatic SNVs and INDELS were 6505 and 5929, respectively. 2064 mutations with a somatic score higher than 0.1 were the high confidence ones that included 963 SNVs and 1101 INDELs which were further classified into 556 insertions, 199 deletions and 364 multiple nucleotide substitutions. Somatic SNVs and INDELs were organized into 4 groups according to their position in the genome:

**Table 1:** Summary of Complete Genomics sequencing results.

Gross mapping yield (Gb)		
	Tumor	222.02
	Normal	234.53
Fully called genome fraction		
	Tumor	95.30%
	Normal	96.40%
SNV Transition/transversion		
	Tumor	2.14591
	Normal	2.14218
SNV total count		
	Tumor	3,096,049
	Normal	3,314,611
Novel SNV^+^		
	Tumor	133,662
	Normal	145,929
High confidence somatic SNV		963
	Non-synonymous	9
	Splicing sites	1
	miRNA loci	0
INDEL^i^ total count		
	Tumor	395,724
	Normal	433,906
Novel INDEL^+^		
	Tumor	67,163
	Normal	74,562
High confidence somatic INDEL		1101
	Coding region	2
	Splicing sites	0
	miRNA loci	0

+ Novel: not previously described in dbSNP131; ^I^INDEL group includes insertion, deletion and substitutions.

#### Tier 1

Mutations with the following characteristics: (n = 12).

- In coding regions causing non-synonymous amino acid changes, in miRNA loci or splicing sites.
- Not previously described in dbSNP131 [13] or in the 1000 Genomes Project [14] or in regions of segmental duplication [15] (reported in the Segmental duplication track of University of California Santa Cruz (UCSC) genome browser http://genome.ucsc.edu/).

#### Tier 2

Mutations affecting UTRs, ncRNA (different from miRNA) and highly conserved regions defined according to the non-repeat masked regions available in UCSC genome browser website (n = 52). Degree of conservation was calculated using phastCons46way tables: phastCons is an algorithm that generates similarity scores from the multiple alignments of 46 vertebrate genomes to the human genome. PhastCons score >500 discriminates highly conserved regions [16]).

#### Tier 3

Mutations affecting non-repeated regions (n = 625).

#### Tier 4

All other mutations (n = 1379).

Tier 1 mutations (10 SNVs and 2 INDELs) were confirmed using Sanger re-sequencing (Figure 1C). The predicted effect on protein structure was estimated using SIFT [17] and PolyPhen2 [18] algorithms and reported in table 2. All the confirmed somatic non-synonymous SNVs, except PPP1R3A^G106A^, were predicted to be deleterious to their respective proteins by at least one method.

**Table 2:** Prediction of mutation effect on protein function.

Gene	Position	Old/new nt	SIFTPrediction	SIFTScore	PolyPhen2Prediction	PolyPhen2 Score
BCOR ^L1067fs^	chrX:39930263-39930263	-TC	Frame shift	–	–	–
FBN3^S1756del^	chr19:8162193–8162195	CTG/−	In frame	–	–	–
LDB3	chr10:88476529	G/T	Splicing site	–	–	–
PCNXL3 ^P327Q^	chr11:65385813	C/A	Tolerated	0.57	Damaging	0.998
PHF15^L569Q^	chr5:133914340	T/A	Tolerated	0.59	Damaging	1
PION^E498G^	chr7:76978720	T/C	Tolerated	0.1	Damaging	0.974
PPP1R3A^G1063A^	chr7:113517959	C/G	Tolerated	0.24	Tolerated	0.001
SFXN3^F82S^	chr10:102795325	T/C	Damaging	0	Damaging	0.996
SRGAP1^R585H^	chr12:64491096	G/A	Damaging	0	Damaging	1
TAF1^R1435C^	chrX:70627923	C/T	Damaging	0	Damaging	1
VN1R5^R193W^	chr1:247419950	C/T	–	–	Damaging	0.986
WDR70^R608H^	chr5:37727093	G/A	Damaging	0	Damaging	1

A coding sequence mutation can damage or preserve the function of the related protein. Several bioinformatics algorithms have been developed to predict the effect of mutations. We used 2 independent methods based on different assumptions. SIFT prediction is based on the degree of conservation of amino acid residues in sequence alignments derived from closely related species. Polyphen2 prediction is based on various sequence and structure-based features of the substitution site.

The expression of the mutated genes was evaluated using transcriptome sequencing. Overall, 199.18×10^6^ reads were generated using a single line of the sequencer’s flow cell. 1.15×10^6^ reads had a mapping quality score below 20 and were excluded from the alignment. The 188×10^6^ reads mapped to the reference genome showed a duplication rate of 36.6%, therefore only 119.02×10^6^ reads were considered for the further RNA sequencing analysis. Transcriptome sequencing confirmed the presence of all mutations in coding sequences, with the exception of LDB3 and PPP1R3A^G106A^ because the genes were not transcribed, and PION^E498G^ and TAF1^R1435C^ because only the wild-type alleles were expressed (Table 3 and Table S1).

**Table 3:** Transcriptome sequencing data of tier 1 mutations.

Mutation	Ensembl ID	Cufflinks FPKM	Low CI	High CI	Mutated allele expression
BCOR ^L1067fs^	ENSG00000183337	18.87	0.00	836.48	90%
FBN3^S1756del^	ENSG00000142449	1.66	1.53	1.79	67%
LDB3	ENSG00000122367	0.00	0.00	0.00	na*
PCNXL3 ^P327Q^	ENSG00000197136	18.96	18.33	19.59	42%
PHF15^L569Q^	ENSG00000043143	37.99	0.00	214.34	59%
PION^E498G^	ENSG00000186088	15.68	0.00	140.89	0%
PPP1R3A^G1063A^	ENSG00000154415	0.00	0.00	0.01	no reads
SFXN3^F82S^	ENSG00000107819	6.01	0.00	13.42	14%
SRGAP1^R585H^	ENSG00000196935	1.29	0.00	27.89	27%
TAF1^R1435C^	ENSG00000147133	12.22	0.00	64.17	0%
VN1R5^R193W^	ENSG00000197617	0.03	0.00	0.06	50%
WDR70^R608H^	ENSG00000082068	25.67	24.21	27.13	34%

The expression of tier1 genes was estimated using Transcriptome sequencing data. For each gene, identified by a unique Ensembl identity (ID) code, the estimation of expression was calculated using Cufflinks and reported as FPKM (number of fragments per kilobase of exon per million fragments mapped: Cufflinks FPKM). Each FPKM value was calculated with a confidence interval ranging between Low CI and High CI values. Mutated allele expression summarizes the percentage of mutated reads covering the mutation site.

### Structural Variations

**Figure 2:**
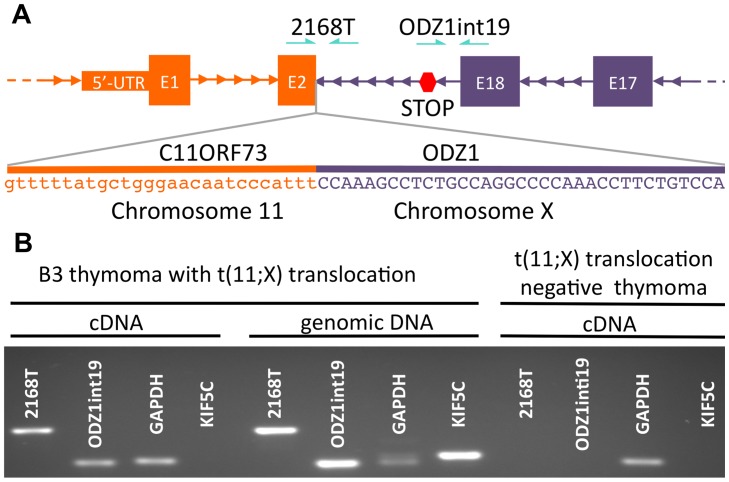
t(11;X) translocation. (A) Schematic illustration of t(11;X); STOP: inframe TAA codon. (B) PCR amplification using primers spanning C11ORF73 exon 2-ODZ1 intron 19 junction (2168T), ODZ1 exon 18-intron 19 junction (ODZ1 int19), GAPDH exons 9 and 10 and introns across exon 3 of an unrelated gene (KIF5C) on genomic and cDNA of the t(11;X)-positive B3 thymoma and a t(11;X)-negative thymoma cDNA. Note that the presence of KIF5C PCR product in the B3 thymoma genomic DNA but not cDNA suggests that the cDNA is free of genomic DNA contamination.

The analysis of whole genome sequencing predicted the presence of 3 structural variations supported by more than 20 discordant paired-end reads with a span size larger than 5 kb [6] (Table 4). However, confirmation of these structural variations demonstrated exclusively the presence of t(11;X) involving the coding region of exon 2 of C11orf73 and intron 19 of ODZ1. The predicted transcript resulted from juxtaposition of the two genes in tail-to-tail (3′-3′) orientation (Figure 2A). RT-PCR demonstrated the presence of the junction transcript in this t(11:X) B3 thymoma but absence in another thymoma without the t(11;X) translocation (Figure 2B). DeFuse [19] and FusionMap [20] algorithms did not identify the fusion transcript in the transcriptome sequencing dataset (Table S2).

**Table 4:** Candidate structural variations identified by complete genome sequencing.

Variation	Left position	Left strand	Right position	Right strand
1	chr11:49883579	−	chrM:16083	+
2	chr11:86017394	+	chrX:123645300	+
3	chr1:158725872	+	chr2:148952671	−

Structural variations such as large deletions, insertions inversions or translocations were predicted using whole genome sequencing data. Structural variations were hypothesized in the presence of a junction sequence: a hybrid sequence composed of two non-adjacent fragment of DNA. In the table are reported the candidate structural variations of this B3 thymoma: the extremity position of the 2 joint sequences and the strand of orientation. Among these candidates only the translocation t(11;X) was confirmed by PCR.

### Copy Number Aberrations

CN estimation from next generation sequencing data agreed with array CGH analysis and revealed CN gain of chromosomes 1q, 5 and 7 and CN loss of chromosomes 3p, 6, 13 and part of chromosome 11q in the autosomal chromosomes (Figure 1C). These data are compatible with those described for B3 thymomas in CGH studies by Zetel A, et al., [5] Girard N, et al. [21] and our group [4]. CGH results confirmed the presence of a large proportion of tumor cells in the sample.

## Discussion

Here we have reported the first genome wide sequencing of a thymoma. Our data showed the mutations (single nucleotide variation and insertion/deletion), copy number aberrations and the translocation of a case of B3 thymoma.

Thymomas are a group of neoplasms with a low to moderate malignant potential. For this reason, thymomas are distinct from thymic carcinomas that are frankly aggressive tumors. The molecular alterations supporting the growth of thymoma cells have not being identified to date. Although, some reports described mutations in thymic carcinomas such as KIT and TP53 mutations, for example [2], only sporadic mutations have been described in thymomas. Despite the less aggressive behavior of thymomas, compared to carcinomas and leukemias, our high-throughput screening revealed a similar mutation rate 0.72/Mb (single nucleotide variations and insertion/deletions) of that observed in neoplasms such as breast cancer (1/Mb) [22] and myelogenous leukemias (0.56/Mb) [23]. However, a higher average number of mutations was reported for lung cancer (8/Mb) and melanoma (up to 111/Mb) [6], [24].

We selected a B3 thymoma for this study because B3 tumors possess an aggressive behavior and frequently necessitate systemic treatments, but yet they maintain a thymic gland structure, unlike thymic carcinomas. The identification of druggable targets in this subset of tumors is of clinical interest. B3 thymomas are frequently diagnosed as locally advanced and a preoperative chemotherapy treatment is often performed in order to increase the resectability rate. The present case well represents this situation being treated with preoperative chemotherapy. Therefore, some of the observed mutations may be introduced by the preoperative treatment, although it is not known how extensively chemotherapy may influence this process.

CGI technology identified 3,314,611 SNVs in the normal genome of this patient. Our results are in line with previous reports describing between 3 and 4.2 million of SNPs when a person’s genome is compared to the reference NCBI genome [25], [26], [27], [28]. Because the reference was built on a Caucasian genome, people with a Caucasian ancestry present a lower number of SNPs, close to 3 million, similarly to this case. The number of SNPs observed in the tumor was 3,096,049. A larger number of SNPs in normal than in tumor DNA was expected because: large genomic regions in the tumor genome have CN losses and tumor specific SNVs were only 0.03% of the SNPs (normal genome vs NCBI reference). Only one allele, of the 2 present in normal DNA, remains in region of heterozygous CN loss. Consequently, regions of CN loss present less SNPs because the SNPs of the lost allele are not represented anymore. Indeed, chromosomes with CN loss had fewer SNPs in tumor than in normal DNA (average difference 24%). On the contrary, in chromosomes without CN loss, the number of SNPs in tumor and normal was similar (average 2% more SNPs in normal with a standard deviation of 2%).

Although SNVs and INDELs are only occasionally described in thymomas, a well-known pattern of CN aberrations characterizes these tumors. Indeed, different histotypes present different CN aberrations, with type A usually displaying a more normal and B3 a more aberrant karyotype [4]. B3 thymomas frequently present CN gains of chromosomes 1, 5, 7, 9 and X and CN losses of chromosomes 6 and 13 [4] [5]. The CN aberrations observed in this case are comparable with those previously described in B3 thymomas. The CN aberrations predicted by CGI data were confirmed by array CGH. The presence of such aberrations demonstrated a high neoplastic cellularity in the tumor sample. This is necessary for the identification of tumor specific events using the adopted pipeline of next generation sequencing data analysis. The observed CN aberrations are arm level CN gain and loss. Each of them affects thousand of gene loci. In the literature the relevance of these large CN alteration events is only marginally characterized. However, the CN loss of chromosome 13 is intriguing because this affects the loci of well-characterized tumor suppressor genes such as RB1 and BRCA2.

The translocation t(11;X) was identified using whole genome sequencing and confirmed using RT-PCR. The breakpoints of this translocation were mapped in the exon 2 of C11orf73 and intron 19 of ODZ1 (TENM1 teneurin transmembrane protein 1). This translocation has not been previously reported. Similarly, translocations and fusion proteins, between C11orf73 or ODZ1 and different partners, were not described. A fusion protein of immunoglobulin heavy chain and ODZ2, was observed in mucosa-associated lymphoid tissue lymphoma [29]. ODZ2 (TENM2 teneurin transmembrane protein 2) is a related gene of teneurin family mapped on chromosome 5. The integration of CN aberrations and structural variations led us to suppose that the translocation t(11;X) was responsible for the CN loss of part of chromosome 11. RNA sequencing was unable to identify a fusion transcript between C11orf73 and ODZ1, using either DeFuse [19] or FusionMap [20] algorithms. This result was expected, because the poly-A tail capture strategy, adopted for mRNA enrichment, was not expected to capture the C11orf73-ODZ1 fusion transcript, since the nature of their tail-to-tail (3′–3′) fusion was doomed to lose the poly-A tails of both genes. Very little is known about the function of C11ORF73, whereas ODZ1 is a type II transmembrane protein belonging to the tenascin family that may function as a cellular signal transducer dimerizing with other members of the teneurin super family [30]. The expressed fusion transcript includes ODZ1 intron 19 sequence, that carries an inframe stop codon, 4 amino acids downstream the last codon encoded by exon 18. The expression of the fusion transcript containing a large intron is supposed to trigger nonsense-mediated decay [31]. Exon junction complexes may encounter the premature stop codon in intron 19 immediately downstream of exon 18 of ODZ1 resulting in mRNA degradation rather than the expression of a truncated form of the protein.

The whole genome sequencing of this B3 thymoma did not identify any mutations of well-characterized cancer genes. Therefore, it was difficult to understand the relevance of the detected mutations. Consequently, it turns out to be a more attractive experience to sequence additional thymoma genomes than ascertain the frequency of mutations exclusively of these genes. Tier 1 mutations were not observed in other tumor types according to the catalogue of somatic mutations in cancer (COSMIC) and were not previously described as SNP in public databases such as dbSNP131 and 1000 Genomes Project. Interestingly, every somatic SNV in tier 1, but PPP1R3A, had a damaging effect on the protein structure as predicted either by SIFT or Polyphen 2. Integrating transcriptome and whole genome sequencing data, it was possible to determine the expression of the mutated alleles of the tier1 genes (Table 3). Neither LDB3 nor PPP1R3A were expressed. LDB3 mutation was localized in a splicing site but RNA sequencing data did not show any mis-spliced transcripts. As reported in gene expression atlas (http://www.ebi.ac.uk/gxa) there was little expression for LDB3 and PPP1R3A in normal thymus. Conversely, PION and TAF1 were expressed in this tumor but the mutated alleles were not observed in the transcriptome data. Interestingly, both these loci were in regions of CN gain: chromosomes 7 and X. The relevance of these mutations remains difficult to ascertain in this tumor. On contrary, the mutated allele of the remaining tier 1 genes was expressed. The function of FBN3, PCNXL3, SFXN3, SRGAP1, VN1R5 and WDR70 have been only marginally evaluated, and their role in the molecular signaling of tumor cells remains largely understudied. According to the literature, PHF15 and BCOR mutations can be related to the neoplastic phenotype. PHF15, known also as JADE2, belongs to a family of more than 100 proteins characterized by the Plant Homo Domain (PHD) finger [32]. Several PHF proteins are localized in the nucleus, and are involved in chromatin-mediated gene regulation. Other members of PHF family are ASH1L, MLL and AIRE. Interestingly, the expression of AIRE, an autoimmune regulatory gene, is commonly down-regulated in thymoma [2]. AIRE expression was not detected using transcriptome sequencing (FPKM 0 95CI: 0-0). The reduced expression of AIRE may play a crucial role in the pathogenesis of the autoimmune paraneoplastic syndromes frequently associated with thymoma [2]. Although AIRE expression was not detected in our sequenced case, the patient did not develop any paraneoplastic autoimmune disease at the time of this report. Although the function of PHF15 is largely unknown, the closely related molecule PHF17 has been shown to have tumor suppressor activity in kidney cancer: PHF17 is normally stabilized by the VHL protein and prevents tumor growth by inducing apoptosis [33]. When over-expressed, PHF17 can increase histone H4 acetylation in vivo [34].

BCOR is a corepressor of several transcription factors, first discovered for its ability to inhibit BCL6 through the BCOR-BCL6 binding domain [35]. Interestingly, BCOR mutated allele was preferentially expressed, accounting for 90% of the RNA sequencing reads (Table 3). This frequency of expression is relevant because the observed mutation is a frameshift insertion possibly inactivating the protein and its corepressor activities. BCOR is a transcription repressor essential for hemopoiesis, mesenchymal stem cell and early embryonic development [36], . Germline BCOR mutations cause Oculofaciocardiodental syndrome, an inherited X-linked syndrome characterized by cardiac defects and dysmorphic appearance [38]. Similar genetic inherited malformations are also observed in the presence of the 22q11.2 deletion that is responsible for a group of syndromes such as the Di George or the velo-cardio-facial syndrome frequently associated with thymic aplasia or hypoplasia [39]. The 22q11.2 deletion syndromes are linked to the loss of TBX1, a gene necessary for normal thymus development [39]. This parallelism may indicate the relevance of BCOR to growth of thymic epithelial cells [39]. Recently, BCOR has been described as mutated in a subset of acute myeloid leukemia patients [40]; about 50% of which present with both BCOR and DNMT3A mutations suggesting a potential cooperation of the two genes, possibly through an epigenetic mechanisms [40]. BCOR was significantly mutated in medulloblastoma such as other nuclear co-repressor (N-CoR) complex genes [41]. BCOR may also facilitate transcriptional repression through interacting with epigenetic regulators such as class I and II HDACs or the polycomb group proteins [40].

The relevance of these mutations is hard to assess due to their absence in the genome of other tumor types and by the insufficient functional characterization of the mutated genes. Therefore, it is important to determine which are the recurrently mutated genes in thymomas to guide the selection of those relevant for the biology of this disease. Perhaps whole exome sequencing is a more cost effective strategy to address this question than a targeted re-sequencing of all the exons of tier 1 genes, allowing the identification of other recurrent non-synonymous mutations in thymic epithelial tumors besides these 12 genes. The present results underline the necessity to identify which are the recurrently mutated genes in thymoma because they may represent useful targets for treatment and for their role in the pathogenesis of the disease. It is also conceivable that genetic alterations other than mutations are of importance in the causation and progression of thymomas, such as epigenetic changes, or miRNA alterations, as described in other tumor types [42]. A broad approach to the study of this rare tumor type may indeed be warranted, similar to the one taken for more common tumor types (e.g. The Cancer Genome Atlas program and Cancer Genome Program at Sanger). Such investigation is ongoing at our institution.

Whole genome sequencing and accompanying transcriptome sequencing describe for the first time the mutations of a thymoma on a genome wide scale and offer an unprecedented view of those events responsible for the growth of these tumors. The absence of mutations affecting the most common cancer genes suggests a possible thymoma specific pattern of mutations indicating the necessity of additional genome wide screenings rather than to determine the mutation frequency of a panel of common cancer genes.

## Supporting Information

Table S1 (XLS)

Table S2 (DOC)

Materials S1 (DOC)
