# Supplementary material for: Whole Genome and Transcriptome Sequencing of a B3 Thymoma
Source: PLoS One. 2013 Apr 5;8(4):e60572. doi: 10.1371/journal.pone.0060572 (PMC3618227; doi:10.1371/journal.pone.0060572)
Supplement: Table S2 — (DOC) [file pone.0060572.s002.doc]

| **Supporting table S2:** predicted fusion genes | | | | | | |
| --- | --- | --- | --- | --- | --- | --- |
| **Fusion #** | **Prediction method** | **Breakpoint 1** | **Breakpoint 2** | **Gene 1** | **Gene 2** | **Confirmed*** |
| 1 | FusionMap | chr12:8542866 | chr8:6914231 | LOC389634 | DEFA5 | no |
| 2 | deFuse | chr3:52275113 | chr3:52284613 | RP5-1157M23.2 | PPM1M | no |
| 3 | deFuse | chrX:133988640 | chr8:58039321 | FAM122C | 5S_rRNA | no |
| 4 | deFuse | chr14:61191066 | chr14:61436671 | SIX4 | MNAT1 | no |
| 5 | deFuse | chr20:4168394 | chr20:4176375 | SMOX | RP4-779E11.3 | no |
| 6 | deFuse | chr17:42153711 | chr17:42201014 | G6PC3 | HDAC5 | no |

* RT-PCR demonstrated the predicted calls were false negative.
