# Supplementary material for: Whole Genome and Transcriptome Sequencing of a B3 Thymoma
Source: PLoS One. 2013 Apr 5;8(4):e60572. doi: 10.1371/journal.pone.0060572 (PMC3618227; doi:10.1371/journal.pone.0060572)
Supplement: Materials S1 — (DOC) [file pone.0060572.s003.doc]

**Supporting Materials**

**Supporting methods:**

**Fusion transcripts from RNA sequencing**

Two previously described algorithms FusionMap and DeFuse were used to predict fusion transcripts from RNA sequencing data.

FusionMap version 2.10.8 was downloaded from authors’ website (http://www.omicsoft.com/fusionmap/ website). According to previous reports, the following FusionMap’s parameters were applied to generate predictions of the fusion transcripts : MinimalFusionAlignmentLength=25, FusionReportCutoff=1and NonCanonicalSpliceJunctionPenalty=4. Candidate fusions were filtered to remove a list of false positive results identified by the authors and available from their website. Only predictions supported by >20 seed reeds were considered.

DeFuse software was downloaded from the author’s website (<http://compbio.bccrc.ca/software/defuse/>) and fusion candidates were calculated and filtered as previously described .

Candidate fusions, generated with both methods, were further filtered. Using Blat tool from USCS ([http://genome.ucsc.edu](http://genome.ucsc.edu/)) we selected only those predicted junctions that showed less than 97% identity to multiple genomic or contig sequences. Candidate fusions were chosen if none of their breakpoints was included in a human chained self alignment or in repeated region: segmental duplications , repeat maskers , interrupted repeats and simple repeat .

**Validation of predicted fusion transcripts**

All the candidates were not validated by RT-PCR. cDNA was generated from patient’s RNA using High Capacity Reverse Transcriptase (Applied Biosystems). Primers, available upon request, were designed on opposite arms of the junction sequence. We included as positive controls: primers in normal GADPH sequence and primers amplifying junction predicted with the same methods in different tumors of unrelated studies. cDNA was amplified by PCR using Taq DNA polymerase (Invitrogen) and Veriti® 96-Well Thermal Cycler (Applied Biosystems). Southern blots of PCR products were run on 1.2% agaraose gels.

**Supporting references.**

1. Ge H, Liu K, Juan T, Fang F, Newman M, et al. (2011) FusionMap: detecting fusion genes from next-generation sequencing data at base-pair resolution. Bioinformatics 27: 1922-1928.

2. McPherson A, Hormozdiari F, Zayed A, Giuliany R, Ha G, et al. (2011) deFuse: an algorithm for gene fusion discovery in tumor RNA-Seq data. PLoS Comput Biol 7: e1001138.

3. Bailey JA, Gu Z, Clark RA, Reinert K, Samonte RV, et al. (2002) Recent segmental duplications in the human genome. Science 297: 1003-1007.

4. Chiaromonte F, Yap VB, Miller W (2002) Scoring pairwise genomic sequence alignments. Pac Symp Biocomput: 115-126.

5. Jurka J (2000) Repbase update: a database and an electronic journal of repetitive elements. Trends Genet 16: 418-420.

6. Benson G (1999) Tandem repeats finder: a program to analyze DNA sequences. Nucleic Acids Res 27: 573-580.
